# Alternative reproductive tactics in snail shell-brooding cichlids diverge in energy reserve allocation

**DOI:** 10.1002/ece3.1495

**Published:** 2015-04-27

**Authors:** Corinna von Kuerthy, Linda Tschirren, Michael Taborsky

**Affiliations:** Behavioural Ecology, Institute of Ecology and Evolution, University of BernWohlenstr. 50a, 3032, Hinterkappelen, Switzerland

**Keywords:** Bourgeois males, capital and income breeding, energy allocation, fixed and plastic parasitic tactics, reserves, storage

## Abstract

Life history theory predicts that the amount of resources allocated to reproduction should maximize an individual's lifetime reproductive success. So far, resource allocation in reproduction has been studied mainly in females. Intraspecific variation of endogenous energy storage and utilization patterns of males has received little attention, although these patterns may vary greatly between individuals pursuing alternative reproductive tactics (ARTs). ARTs are characterized by systematic variation of behavioral, physiological, and often morphological traits among same-sex conspecifics. Some individuals may rely on previously accumulated reserves, because of limited foraging opportunities during reproduction. Others may be able to continue foraging during reproduction, thus relying on reserves to a lesser extent. We therefore predicted that, if male tactics involve such divergent limitations and trade-offs within a species, ARTs should correspondingly differ in energy reserve allocation and utilization. To test this prediction, we studied short-term and long-term reserve storage patterns of males in the shell-brooding cichlid *Lamprologus callipterus*. In this species, bourgeois males investing in territory defense, courtship, and guarding of broods coexist with two distinct parasitic male tactics: (1) opportunistic sneaker males attempting to fertilize eggs by releasing sperm into the shell opening when a female is spawning; and (2) specialized dwarf males attempting to enter the shell past the spawning female to fertilize eggs from inside the shell. Sneaker males differed from other male types by showing the highest amount of accumulated short-term and long-term fat stores, apparently anticipating their upcoming adoption of the nest male status. In contrast, nest males depleted previously accumulated energy reserves with increasing nest holding period, as they invest heavily into costly reproductive behaviors while not taking up any food. This conforms to a capital breeder strategy. Dwarf males did not accumulate long-term fat stores at all, which they can afford due to their small behavioral effort during reproduction and their continued feeding activity, conforming to an income breeder strategy. Our data confirm that the resource storage patterns of males pursuing ARTs can diverge substantially, which adds to our understanding of the coexistence and maintenance of alternative reproductive patterns within species.

## Introduction

Within populations, individuals often differ in the way they deal with social and ecological challenges, because competition for resources and reproduction can select for divergent coping strategies (Taborsky 1994; Sih et al. 2004; Taborsky and Brockmann 2010). This may lead to remarkable phenotype polymorphisms that are associated with alternative reproductive tactics (ARTs; [Gross 1996; Brockmann 2001; Oliveira et al. 2008]). ARTs are characterized by bimodal or multimodal distributions of behavioral, physiological, and sometimes morphological traits within same-sex conspecifics, which result from disruptive sexual selection, typically in males (Taborsky et al. 2008; Taborsky and Brockmann 2010). At the behavioral level, large “bourgeois” males usually monopolize resources to attract mates, which creates opportunities for male competitors to exploit their effort (Taborsky 1994, 1998, 2001; Neff et al. 2003). While males of the bourgeois pathway may invest more into growth (Wirtz-Ocaňa et al. 2013), conspicuous body ornaments (Neat et al. 2003; Candolin and Wong 2008), extended phenotypes (Schaedelin and Taborsky 2006, 2009), or weaponry (Tschernavin 1938), parasitic males may instead benefit from a smaller and inconspicuous appearance (Taborsky 1994) and from investing into testis size and sperm production (Gage et al. 1995; Taborsky 1998; Neff et al. 2003). Resource allocation and reproductive investment patterns of individuals specializing in either reproductive monopolization or parasitic behavior can diverge substantially, causing different limitations and trade-offs (Dominey 1981; Neat et al. 2003; Schütz et al. 2010; Schradin and Lindholm 2011).

The ability to store energy in order to compensate for resource deficits during development and reproduction is an important component of life history variation (Stearns 1989; Jonsson 1997; Houston et al. 2007). Depending on the size, age, and the pursued reproductive tactic, individuals may greatly differ in their temporal distribution of resource acquisition and use (Jonsson 1997; Taborsky 2006). In teleosts, for instance, immature individuals typically allocate most available energy to speed up growth, because large size reduces their vulnerability to predators (the “bigger is better” hypothesis) (Miller et al. 1988; Taborsky et al. 2003). With increasing age, individuals usually reduce their investment in growth (Wirtz-Ocaňa et al. 2013) and instead expand energy for behavioral, morphological, and physiological features in preparation of reproduction.

Fish show a wide spectrum of energy allocation strategies ranging from capital breeding, as exhibited by guppies (Poecilia) and swordtails (Xiphophorus), to income breeding as observed in killifish (Stearns 1992). Capital breeders use energy for reproduction that they have gained earlier and stored, whereas income breeders use energy gained by feeding during reproduction (Bonnet et al. 1998; Andersen et al. 2000). To date, the concept of capital and income breeding has been applied mainly to females (Doughty and Shine 1997; Lourdais et al. 2002; Broussard et al. 2005; Houston et al. 2007), where the focus is usually on the period before egg laying or birth of young (Doughty and Shine 1997; Bonnet et al. 1998), or in brood-caring species on the period between birth and weaning (Boyd 2000). Few studies have focused on the variation of energy allocation patterns of males within a species (Mysterud et al. 2005), which is of particular interest when males pursue ARTs (Schütz et al. 2010). Bourgeois males, for instance, may adopt a capital-breeder strategy (Jonsson 1997). For defense of resources and the monopolization of mates and a breeding site, they may rely on previously accumulated energy stores, as the opportunities to forage during their reproductive period may be limited. In contrast, parasitic males can often afford to acquire resources during their reproductive period (Neff 2003) and may thereby act as income breeders (Andersen et al. 2000; Quetglas et al. 2011). Species with ARTs therefore represent a suitable test case for predictions of life history theory regarding the dynamics of endogenous energy storage and utilization prior and during reproduction.

**Figure 1:**
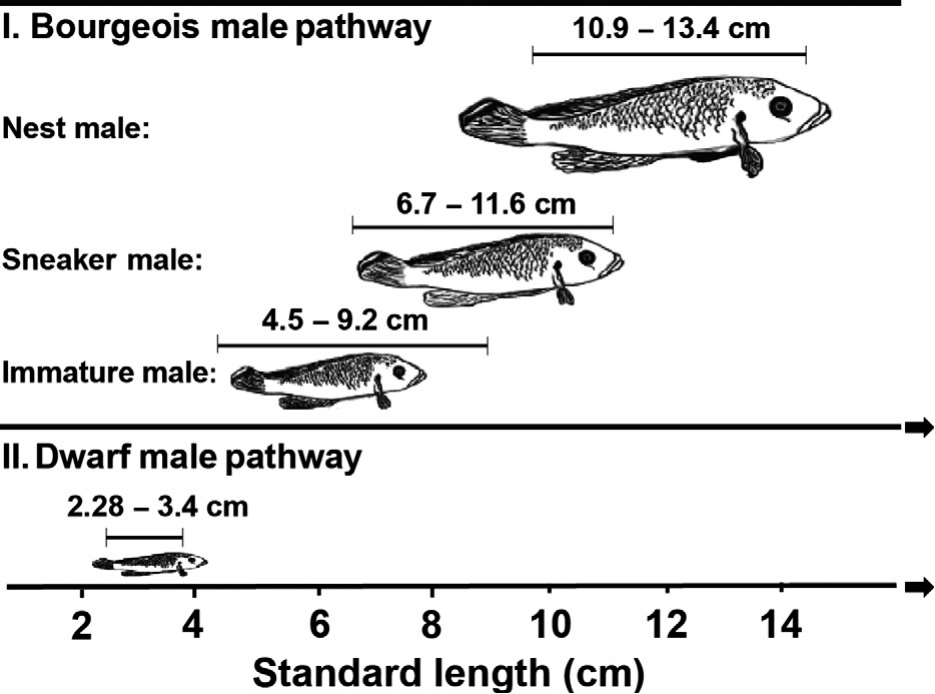
Two genetically distinct pathways in *Lamprologus callipterus* males: I. Bourgeois male pathway with immatures, mature sneaker males, and nest males. II. Dwarf male pathway with adult dwarf male. Immature dwarf males were not included in this study, because they cannot be unequivocally identified and collected in the field. Size ranges of all males represent standard lengths (cm) of individuals collected in this study.

*Lamprologus callipterus* is a polygynous, biparental cichlid from Lake Tanganyika. Males of this species show one of two alternative life history pathways determined by a Mendelian genetic polymorphism (Wirtz-Ocana et al. 2014). Large bourgeois males (“Nest males”: Fig.1) collect empty snail shells (mainly *Neothauma tanganyicense*) and defend them against other males and predators, thereby providing protection to females breeding inside these shells (resource defense polygyny; (Emlen and Oring 1977; Sato 1994; Sato et al. 2004)).

The nest holding period (NHP), that is, the time a nest male monopolizes and defends a nest, can differ extremely among males (Sato 1994), which is strongly influenced by male body condition (Schütz et al. 2010). During this period, nest males are regularly challenged by the interference of males pursuing one of two alternative mating tactics, which attempt to parasitize the high reproductive investment of nest males (Taborsky 1998, 2001; Sato et al. 2004). Parasitic sneaker males try to steal fertilizations from the nest owner by occasionally darting into a nest male's territory during spawning, which can last for several hours, as the female releases one egg at a time with intervals of several minutes between subsequent eggs (Schütz et al. 2012). After passing a threshold size of ∼9 cm (Schütz and Taborsky 2005), these males may switch from sneaker to nest male status and attempt to hold a territory themselves (see Fig.1).

The genetically fixed dwarf male tactic constitutes a different life history pathway (Fig.1; (Taborsky 1998; Sato et al. 2004; Taborsky 2008). Parasitic dwarf males make up only 2.4% of nest male mass on average, and they show highly specialized mating behavior (Sato et al. 2004); they attempt to steal fertilizations from territory owners by wriggling past a spawning female into the tip of the shell, from where they may fertilize the majority of the eggs (Wirtz-Ocana et al. 2014). In nature, dwarf males have been found to participate in 5% of 120 haphazardly surveyed broods (Wirtz-Ocana et al. 2014). Dwarf males consequently need to halt growth at a certain body size, which means that they can invest all surplus energy into gonads and current reproduction (Schütz et al. 2010; Wirtz-Ocaňa et al. 2013). They also should not accumulate extensive energy stores, as they benefit from a small and slim body when attempting to wriggle into the shell past the female (Sato et al. 2004).

Here, we aim to compare the dynamics of storage and utilization of endogenous lipid reserves between the three male types of *L. callipterus*. We hypothesize that immature males should prioritize growth and hence should hardly accumulate fat stores (Miller et al. 1988). Parasitic sneaker males pursue the bourgeois male life history pathway and hence should build up energy stores to prepare for reproduction as a bourgeois nest owner. Therefore, we predict peak levels of fat stores for individuals that are close to the switch point from sneaker to nest male status, to be prepared for the time of starvation, and high reproductive investment when holding a nest.

Nest males do not forage during their nest holding period (NHP) (Schütz et al. 2010); hence, we predict that due to their high reproductive investment and associated fasting, fat stores should decline in the course of holding a nest. Therefore, any haphazard sampling of nest males in the field should reveal high variance in the proportion of body fat, reflecting the spectrum of reserve states ranging from full energy stores at the beginning of the NHP to largely depleted fat stores at its end. The actual length of the NHP may be influenced by the activity pattern of a nest male during this period. In principle, there are two possibilities how nest males could cope with their dwindling energy reserves; (1) they might reduce activity with increasing NHP to save energy and keep the nest longer; or (2) they might keep up high activity levels to maximize reproductive success while keeping the nest, until a threshold is reached where they cannot continue to defend the nest successfully and therefore leave it abruptly.

In contrast, based on behavioral observations, Schütz et al. (2010) suggested that males of the genetically fixed dwarf male tactic are income breeders. Whereas small dwarf males might benefit from accumulating some fat as this will not prevent them to enter shells by passing spawning females, larger dwarf males might suffer from fat stores that may impede their wriggling past a spawning female inside a narrow shell. Accordingly, we predict that dwarf males store only little fat and avoid long-term energy stores and that the accumulation of energy reserves should decline with increasing age and body size of dwarf males, opposite to the pattern of sneaker males.

In fish, lipids can be stored in and among several organs rather than in one principal depot (liver, muscle, peritoneum; Sheridan 1988). Short-term energy reserves are often stored in the liver and used during the initial stages of starvation, whereas energy stored in the muscles is often utilized at a later phase of starvation (Collins and Anderson 1995). Visceral fat depots stored in the body cavity are serving long-term energy storage, as for instance revealed in rainbow trout (Jezierska et al. 1982). Although the sequence and amount of energy depletion of different types of fat depots may differ among teleost species, visceral fat is typically mobilized at a later point in time than other fat stores, particularly those in the liver (Collins and Anderson 1995; Rios et al. 2006).

The complex breeding system in *L. callipterus* offers unique opportunities to compare within one species the dynamics of accumulation and depletion of energy stores in males pursuing highly divergent, fixed, and flexible ARTs.

## Materials and Methods

### Nest males

We studied *L. callipterus* at Wonzye Point near Mpulungu, at the southern end of Lake Tanganyika, Zambia (lat 8°45.5′S, long 31°06.1′ E), from October to December 2009. A total of 17 nest males were individually marked upon first detection on a nest, using visible implant elastomer tags (Northwest Marine Technology, Inc.). All nests were checked daily by scuba diving to observe focal nest males, identify newly occupied nests in the study area, and to detect nest takeovers by new males. Each new nest male was haphazardly assigned to a previously and randomly chosen number of nest holding days between 1 and 33 days, which was hence its predetermined NHP. Thirty-three days is the mean natural nest holding period in *L. callipterus* (Schütz et al. 2010). As multiple energy measures were not possible, this approach allowed us to measure and compare energy stores of nest males with different nest holding times, to check for a possible depletion of nest male reserves during reproduction. For this purpose, the focal nest males were collected from their nest after their assigned NHP for body composition analysis. Of the 17 nest holding males found at Wonzye Point in 2009, 12 nest males could be thus collected, while four males were replaced by new nest males prior to their allocated collection time, and one male was damaged during transport and therefore could not be analyzed.

### Activity patterns during the nest holding period

We recorded the behavior of focal nest males during their NHP in order to obtain information about possible changes in activity patterns in the course of holding a nest. The behavior was monitored using a handheld computer in a waterproof housing, equipped with the software program Observer 5.0 (Noldus, Wageningen, the Netherlands). Focal nest males were observed for 7 min twice a day (morning and afternoon) for their entire NHP, while recording the following behavioral categories: (1) inactive: when the nest male remained immobile aboveground or was sitting on the bottom. (2) active: when the nest male was (a) foraging, (b) courting, (c) spawning, (d) aggressive, or (e) exploring (i.e., leaving the nest for unknown activities). Our aim was to conduct behavioral observations of each individual on all days of their experimentally assigned NHP. However, some of the observations were missing due to unexpected incidents (e.g., thunderstorms).

### Immature and parasitic males

To compare the energy reserve management among different male types, we also haphazardly collected 10 immature individuals, 10 potential sneaker males, and three dwarf males at Wonzye Point, plus five additional dwarf males at Kasakalawe, a location ∼7.8 km from Wonzy Point. Immature males are usually roaming about in groups to search for food, whereas sneaker males either roam about in groups as well, or stay in the proximity of a nest male's territory where they may attempt to enter a nest to steal fertilizations. Dwarf males are generally harder to find because they cannot be identified unequivocally by their body size and morphology. Only their behavior when trying to enter a nest provides clear information about their tactic. We validated the assignment of all collected males to different tactics by the states of their testes. For collection, all males were first anaesthetized and finally killed with an overdose of MS222 (3-aminobenzoic acid ethyl ester, Sigma-Aldrich Chemie GmbH, Buchs SG, Switzerland). The standard length (SL to the nearest 0, 1 mm) and body mass (BM, 0.1 mg accuracy) of all fish was measured shortly after collection.

### Evisceral and visceral energy reserves

Total fat stores of collected individuals were separated into two different categories: (1) evisceral fat including all fat stored in the liver and muscles, extracted from the whole-body carcasses; and (2) pure visceral fat stored within the body cavity (peritoneum). The body cavity of all collected males was opened, and all visceral fat deposits were carefully collected with tweezers and weighed to the nearest 0.001 mg with a high-precision electronic balance. Visceral fat was stored in air-tight tubes, while the body carcasses, excluding the testes, were air-dried. Both fractions were then frozen and dried again in the warming cabinet prior to fat extractions in the laboratory. The two fat categories were extracted separately using ∼95% n-pentane as fat solvent (Merck AG, Zug, Switzerland.) and Soxhlet extractions (Sawicka-Kapusta 1975). After extraction, fat was weighed to the nearest 0.001 mg with a high-precision electronic scale. One sneaker male was lost during fat extraction. Of all remaining immature individuals (*N* - 10), sneaker males (*N* - 9), nest males (*N* - 12), and dwarf males (*N* - 8), we compared the amount of total fat stores in percent of body mass, the variance of total fat stores, and the two separate fat categories, evisceral fat and visceral fat.

From the 12 extracted nest males, nine males could be used to test for a negative correlation of fat reserves with NHP. The other three individuals had switched nests and disappeared for several days before coming back to their initial nest. As we have no information about these males during their absence (i.e., whether they were feeding to recover their energy stores), we excluded them from this analysis of the correlation of energy stores and NHP, but not from general comparison of total fat stores between different male types.

### Data analysis

Nonparametric Kruskal–Wallis ANOVA and Mann–Whitney *U-*tests (R-package “coin”; [Hothorn et al. 2006]) were performed to compare total, evisceral, and visceral energy stores among male types. Due to multiple comparisons, we used the Holm correction to obtain adjusted *P*-values and to control for the family-wise Type I error (Holm, 1979). Based on the assumption that fat deposits in large dwarf males can impede successful wriggling past a spawning female (Sato et al. 2004), we tested with two-tailed Pearson's correlation analysis the prediction that energy stores in mature dwarf males decrease with increasing body size. As the data were not normally distributed, they were log-transformed before performing Pearson's correlation analysis (R-package “car”; Fox & Weisberg 2011). To test the prediction that immature males invest surplus energy primarily into growth when small (according to the “bigger is better hypothesis”; [Miller et al. 1988]), but increasingly accumulate fat reserves with increasing size (i.e., when approaching maturation) to have reserves available for reproduction, we used two-tailed Pearson's correlation analysis. For sneakers and nest males, we did not expect fat stores to be correlated with body size, as energy reserves should primarily depend on the reproductive state and the upcoming or ongoing nest defense. Therefore, to test for potential size effects on fat storage in these male tactics, two-tailed Pearson's correlation analyses were used.

To test the prediction that total fat reserves of nest males decline with NHP, we used one-tailed Pearson's correlation analysis. In order to identify possible differences between male tactics in the variance of total fat in percentage of body mass, we used Levene's tests for single comparisons of male types.

To test for potential changes of behavior patterns of nest males during their NHP, the average amount of time (in seconds) of the daily behavioral observations was calculated for total activity and for each of the single recorded behaviors (aggression, courting, spawning, feeding, and exploring). We modelled total log-transformed or square-root-transformed data of total activity or single behaviors during NHP with linear mixed-effects models (LMEs) using the R-package “lme4” (Bates 2005), including NHP as a fixed effect and fish identity as random effect in all models. The model was fitted with restricted maximum likelihood (REML). All statistical analyses were performed using R 3.0.2 (R Development Core Team 2009).

## Results

### Total energy stores

**Figure 2:**
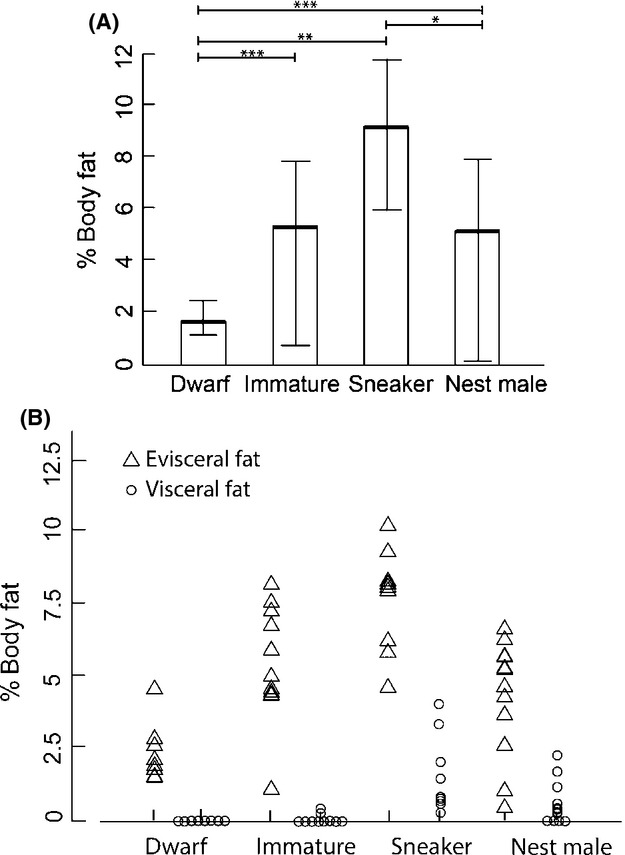
(A) Total fat stores (including evisceral fat and visceral fat) in percent of body mass for dwarf males (*N* - 8), immature males (*N* - 10), sneaker males (*N* - 9), and nest males (*N* - 12; medians and quartiles, significant differences are marked with asterisks; see Table1). (B) Percent evisceral (triangles) and visceral (circles) body fat of dwarf males (*N* - 8), immature males (*N* - 10), sneaker males (*N* - 9), and nest males (*N* - 12). Values superimposed on each other were slightly relocated along the abscissae for better visibility.

Total fat (including evisceral fat + visceral fat), measured as percent of body mass, differed significantly between different male types collected in the field (Table1A: Kruskal–Wallis ANOVA, H(3) - 22.01, *P* < 0.001; Fig.2A). Sneaker males showed the largest proportion of total accumulated fat stores among all male types, which differed significantly from dwarf males and nest males (Table1B, Fig.2A). Nest males and immature individuals had a significantly higher proportion of total fat than dwarf males (Table1B, Fig.2A).

**Table 1:** Comparison of total fat stores (including evisceral fat and visceral fat) in percent of the body mass among different male types in *L. callipterus* (nest male - Nm (*N* - 12), sneaker male - Sn (*N* - 9), dwarf male - Dw (*N* - 8), immatures - Im (*N* - 10)) using Kruskal–Wallis ANOVAs (A) and Mann–Whitney *U-*tests (B). Due to multiple comparisons, we used the Holm correction to control for family-wise Type I error. Significant differences are marked in bold; nonsignificant trends are underlined

(A)			
Fat category	Chi-square	df	*P*-value
Total fat	22.007	3	<0.0001
Evisceral fat	19.304	3	<0.001
Visceral fat	26.108	3	<0.0001

(B)			
Comparison male types	% Body fat adjusted *P*-value	% Evisceral fat adjusted *P*-value	% Visceral fat adjusted *P*-value
Nm < Sn	**0.025**	**0.007**	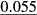
Nm > Dw	**<0.001**	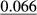	**0.005**
Nm - Im	0.227	0.180	**0.015**
Sn > Dw	**0.006**	**<0.001**	**0.002**
Sn > Im	0.276	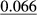	**0.001**
Dw < Im	**0.001**	**0.034**	0.222

The variance of accumulated total fat differed significantly among male types (Levene's test: *F* - 3.184, df - 3, *P *- 0.036; Fig. S1). Pairwise comparisons demonstrated that nest males showed the largest variance of proportions in total fat stores, which differed significantly from dwarf males, but not from sneakers and immature individuals. Sneaker males also showed significantly higher variance of total accumulated fat stores than dwarf males, while immature males differed from dwarf males only marginally (Table2, Fig. S1).

**Table 2:** Comparison of the variances of accumulated total fat stores in percent of body mass of different male types in *L. callipterus*, using Levene's tests. Significantly different variances are marked in bold, a nonsignificant trend is underlined. Symbols in the table represent trends

Groups	F	df	p
Nm - Sn	0.289	1	0.597
Nm > Dw	10.02	1	**0.005**
Nm > Im	0.848	1	0.368
Dw < Im	4.36	1	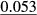
Sn > Dw	7.847	1	**0.013**
Sn - Im	0.155	1	0.698

### Evisceral and visceral fat reserves

Evisceral fat stores in percent of body mass differed significantly among all male types (Table1A: Kruskal–Wallis ANOVA, H(3) - 19.304, df - 3, *P* -< 0.001). Pairwise Mann–Whitney *U-*tests revealed that evisceral fat stores of nest males were significantly smaller than those of sneaker males. The latter stored the greatest amount of evisceral fat, and nest males tended to store more evisceral fat than dwarf males (Table1B).

**Figure 3:**
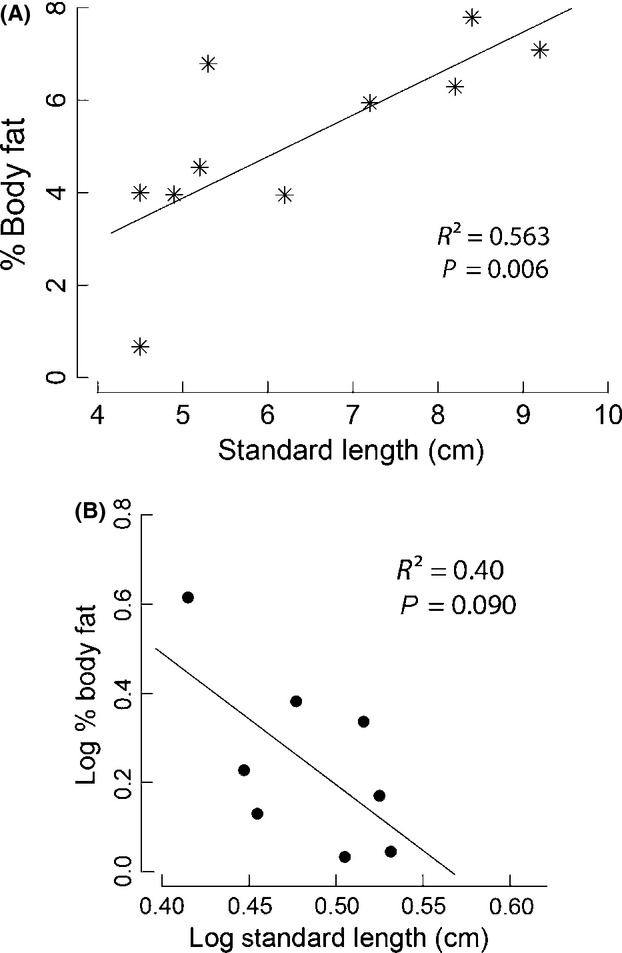
Relationship of total body fat stores (including evisceral fat and visceral fat), in percent of body mass, with standard length (cm) of (A) immature males (*N* - 10) and (B) dwarf males (*N* - 8).

Visceral fat stores (Fig.3B) also differed significantly among male types (Table1A: Kruskal–Wallis ANOVA, H(3) - 26.108, *P *< 0.001). Again, sneaker males showed the greatest visceral fat reserves of all male types (Table1B), and nest males also had larger visceral fat deposits than dwarf and immature males. No dwarf male and only two of ten immature individuals had accumulated any visceral fat (Fig.2B).

### Body size effects

In immatures, total fat reserves in percent of body mass (Mean - 5.10%, SD - 2.11%) correlated positively (Pearson's correlation analysis: *P *- 0.012, *R*² - 0.56, Fig.3A) with body size (Mean - 6.36 cm, SD - 1.76 cm).

Dwarf males showed a nonsignificant negative correlation (Pearson's correlation analysis: *P *- 0.09, *R*² - 0.40, Fig.3B) of percent body fat (Mean - 1.93%, SD - 1.0%) with standard length (Mean - 3.06 cm, SD - 0.29 cm). No correlation of total fat stores and standard length was found in sneakers and nest males (supporting information Figs. S2 and S3).

### Energy depletion and activity of nest males

**Figure 4:**
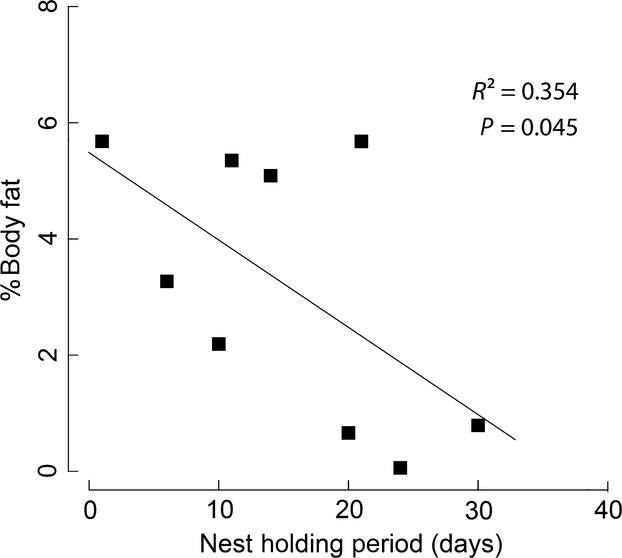
Fat depletion in percent of body mass over the nest holding period. The graph shows the percent of total body fat (including evisceral fat and visceral fat) of different nest males (*N* - 9) on their experimentally determined last day of their nest holding period.

As predicted, the total fat stores of nest males (Mean - 3.94%, SD - 2.49%) declined with time during their NHP (Pearson's correlation analysis: *P *- 0.045, *R*² - 0.35, Fig.4). Total activity of focal nest males did not change with time during their NHP (LME, *N* - 8, *t* - −0.455, *P *- 0.642). Also, none of the observed behaviors (foraging, courting, spawning, aggression, and exploring the area outside the nest) seemed to vary systematically with the time passed since the start of holding a nest (Table3).

**Table 3:** The relationship between the course of the nest holding period (days), total activity (sec), and single behaviors (aggression, courting, spawning, feeding, exploring (sec)) of focal nest males (*N* - 12) tested with linear mixed-effects models (LME)

Response variable	Estimates	Fixed factor	*t*-value	*P*-value
Sqr_Activity(s)	−0.007	NHP	−0.023	0.982
logAggression (s)	−8.730	NHP	−1.272	0.239
logCourting (s)	0.108	NHP	0.565	0.647
logSpawning (s)	−3141	NHP	−0.895	0.367
logFeeding (s)	0.049	NHP	0.669	0.500
logExploring (s)	0.225	NHP	0.326	0.768
logPassive (s)	−0.081	NHP	−1.10	0.267

## Discussion

As predicted by the existence of divergent limitations and trade-offs, different male types of *L. callipterus* vary significantly in reserve accumulation. The separation of fat fractions into visceral fat and evisceral fat (body carcasses without gonads) provided insight into the importance of short- and long-term energy stores for different male types. On the bourgeois male life history pathway, immature individuals accumulate considerable evisceral fat stores during development, but they store hardly any visceral fat. Importantly, the fat reserves of immature individuals rise with increasing body size, which is consistent with the hypothesis that small individuals should use surplus energy mainly for growth (Miller et al. 1988; Meekan et al. 2006) and not for establishing long-term energy stores. Body size affects survival probabilities in *L. callipterus* (Schütz et al. 2006). The fat storage pattern of immature males of *L. callipterus* is in accordance with results from three species of reef fish showing that in small and nonreproductive individuals, no visceral fat was accumulated, while larger individuals did so, most likely also in preparation for reproduction (Fowler 1991).

Sneaker males showed the highest amount of both short- and long-term fat stores among all male types. Total fat stores (including evisceral fat and visceral fat) did not relate to body size, indicating that fat stores of sneakers might depend rather on the reproductive state of individual males, and presumably on their investment into gonads. Consistent with this idea is a nonsignificant positive correlation (*P *- 0.081) between gonad mass and percent total fat reserves in sneaker males (own unpublished data). A correlation between fat metabolism and gonad development was also demonstrated, for instance, in the teleost *Chaetodon rainfordi* (Fowler 1991).

Some sneaker males caught in our study were already in a size range in which they could have defended a nest by themselves (Sato et al. 2004; Schütz and Taborsky 2005). They might have been close to switching from sneaker to nest male status, as they had accumulated large amounts of fat reserves, especially of the long-term visceral fat fraction. This is probably a precondition for founding a nest. The switch from sneaker to territorial male status has been shown to be size and/or condition dependent also in other species with ARTs (Heckel & von Helversen 2002; Oliveira et al. 2002). However, to our knowledge, data on the corresponding allocation and utilization of energy stores have yet been missing.

Our results show that nest male total fat reserves (including evisceral fat and visceral fat) decline during the course of holding a nest, which ultimately may be responsible for the termination of the NHP. Bourgeois nest males showed the largest variance in the distribution of total fat stores in percent body mass among all male types. This reflects the existence of large fat reserves when nest males found a nest, and the depletion of these energy stores during starvation is also caused by high activity levels associated with holding a nest. Interestingly, the depletion of nest male fat stores applied to both fat categories similarly (statistically significant only for total fat stores; Fig.4). In other fish species, visceral fat has been shown to be a major fat depot for reproduction and endurance of long-term starvation. In golden perch, for instance, mobilization of visceral fat bodies occurred between 30 and 60 days of food deprivation, representing long-term fat stores in this species (Collins and Anderson 1995). In the traira (*Hoplias malabaricus*), visceral fat was also consumed gradually during starvation, being exhausted only after a period of 180 days (Rios et al. 2006). A significant decrease in the visceral fat fraction during starvation has been shown also in the ballan wrasse *Labrus bergylta* (Villegas-Ríos et al. 2014).

Energy depletion has been shown to be the major force for giving up a nest or leaving the offspring in other taxa (e.g., Emperor Pinguins; Le Maho, 1983). We expected that due to the diminishing energy reserves, nest males reduce their activities with increasing NHP. However, no relationships between the course of the NHP and general activity or any specific behavior patterns were found. Nest males with a long NHP were still very active, if conspecific male competitors or potential mates arrived at the nest site. Apparently, nest males keep up maximum performance until their energy reserves are depleted, thereby presumably pursuing an “all or nothing” strategy. The large long-term fat stores in sneaker males that have not yet started to defend a nest, and the reserve depletion in nest males during their NHP both confirm the pattern of “capital breeding” (Jonsson 1997) in *L. callipterus* males pursuing the bourgeois tactic (Schütz et al. 2010).

Whereas nest males starve while defending a nest, dwarf males spend ∼20% of their time foraging when reproductively active (Schütz et al. 2010)**.** Additionally, due to the lack of investment into costly reproductive behaviors such as nest building, courtship, and defense, we predicted that dwarf males do not accumulate energy stores, as they appear to perform an income breeder strategy (Schütz et al. 2010). Our data reveal that dwarf males indeed keep lower total fat reserves (dwarf males only had evisceral fat) than all other types of males, and no long-term visceral fat stores at all. Dwarf male reproductive success appears to be primarily limited by the difficulty to enter a shell and wriggle past a spawning female (Sato et al. 2004). The storage of surplus fat could impede the success of this tactic. Additionally, parasitic males suffer from a higher degree of sperm competition than bourgeois males (Gross 1982; Parker 1984), which predicts that dwarf males should prioritize investment into gonads instead of accumulating energy stores. This was confirmed by a comparison of gonadosomatic indices of nest males and dwarf males in *L. callipterus*; the latter exceeded nest males more than fivefold (Sato et al. 2004). Also in other species with ARTs, parasitic males have larger testes relative to their body size than territorial males (e.g., bluegill sunfish; Neff et al. 2003). There was a nonsignificant trend for the amount of fat stored by dwarf males (dwarf males only had evisceral fat) to be correlated with body size, which might suggest that especially relatively large (i.e., old) dwarf males do best by investing surplus energy into gonads instead of accumulating reserves for the rest of their short lives (Rijneveld 2002). This conforms with other species, where older males apparently invest more heavily into testes than younger competitors (Birkhead et al. 1997).

In conclusion, our study illustrates that males pursuing ARTs may differ significantly in short- and long-term fat reserve accumulation and utilization. These energy storage patterns are important correlates of life history variation (Jonsson 1997) and represent crucial components of allocation decisions in species with alternative reproductive tactics.

## Supporting Information

**Figure S1.** Variance of total fat (including evisceral fat and visceral fat) measured in percent of body mass in relation to standard length (cm), for different male types of *L. callipterus* collected in this study: Nest males (squares, *N* - 12), sneaker males (triangles, *N* - 9), immature males (pentagons, *N* - 10), and dwarf males (circles, *N* - 8). **Figure S2.** Relationship of total fat stores (Mean - 9.32%, SD - 2.17%) of sneaker males (*N* - 9) measured in percent of body mass with body size (Mean - 10.37 cm, SD - 1.02 cm). Pearson's correlation analysis: *P *- 0.84, *R*² - 0.006). **Figure S3.** Relationship of total fat stores (Mean - 3.94%, 2.49%) of nest males (*N* - 12), measured in percent of body mass with body size (Mean - 12.1 cm, SD - 0.67 cm). Pearson's correlation analysis: *P *- 0.6672, *R*² -  0.019).
