# Supplementary material for: Alternative reproductive tactics in snail shell-brooding cichlids diverge in energy reserve allocation
Source: Ecol Evol. 2015 Apr 27;5(10):2060–9. doi: 10.1002/ece3.1495 (PMC4449759; doi:10.1002/ece3.1495)
Supplement: Supplementary file 1 [file ece30005-2060-sd1.docx]

**Supporting Information - Ms_Corinna von Kürthy**

**Fig. 5**

**
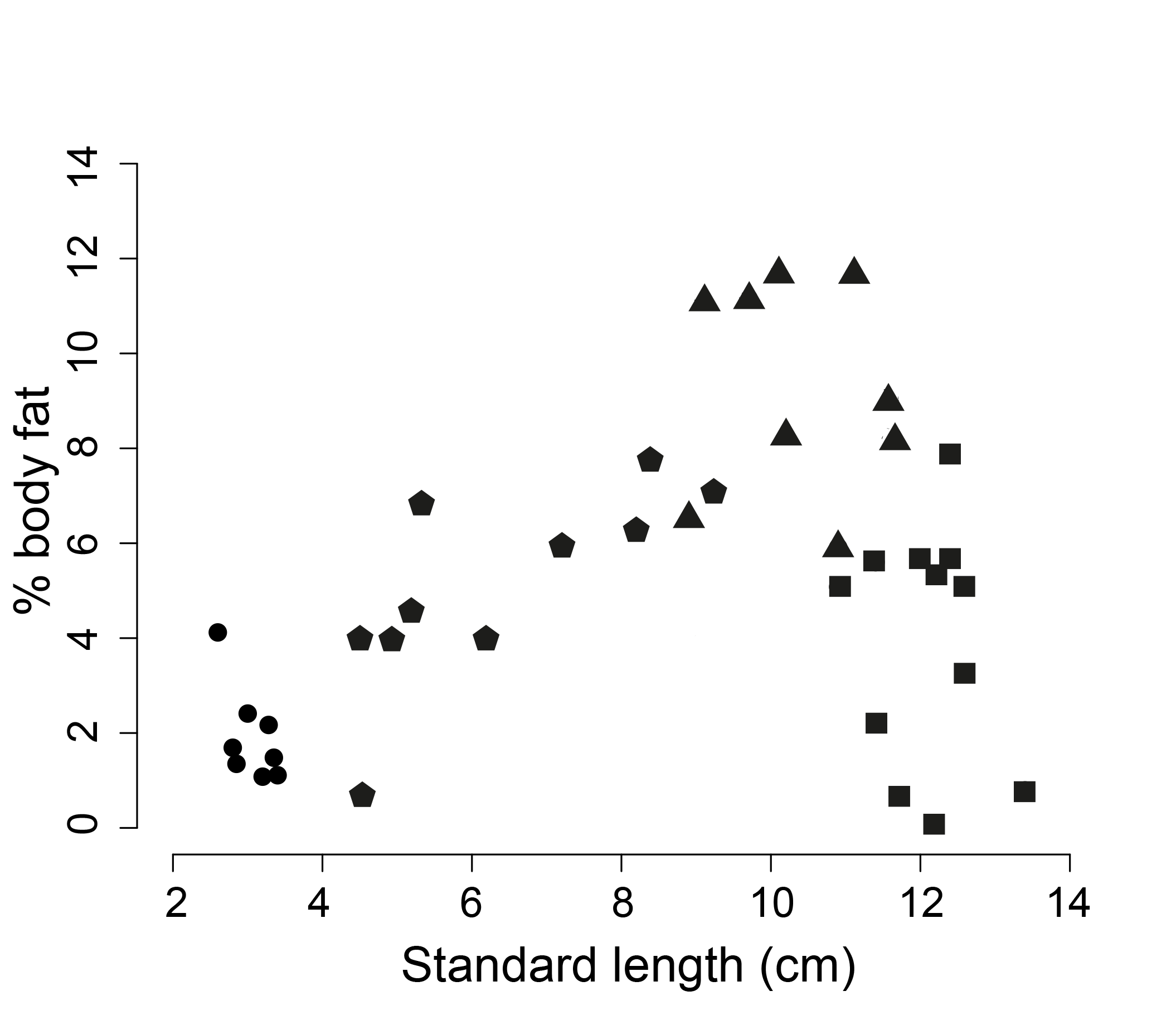
**

**Fig. S1:** Variance of total fat (including evisceral and visceral fat) measured in percent of body mass in relation to standard length (cm), for different male types of *L. callipterus* collected in this study: Nest males (squares, N=12), sneaker males (triangles, N=9), immature males (pentagon, N=10) and dwarf males (circles, N=8).

**Fig.6**

**
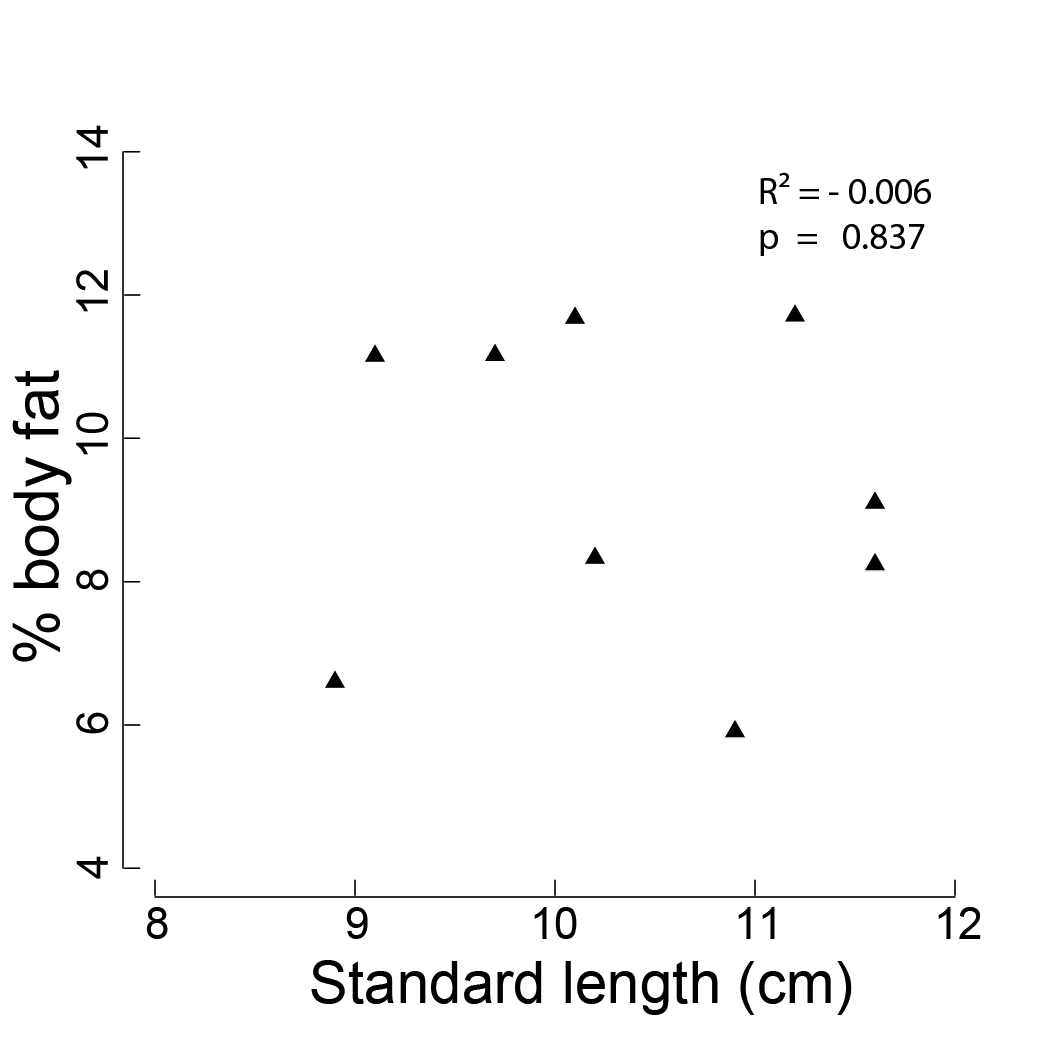
**

**Fig. S2:** Relationship of total fat stores (including evisceral and visceral fat) of sneaker males (N=9) measured in percent of body mass with body size (standard length; cm).

**Fig.7**

**
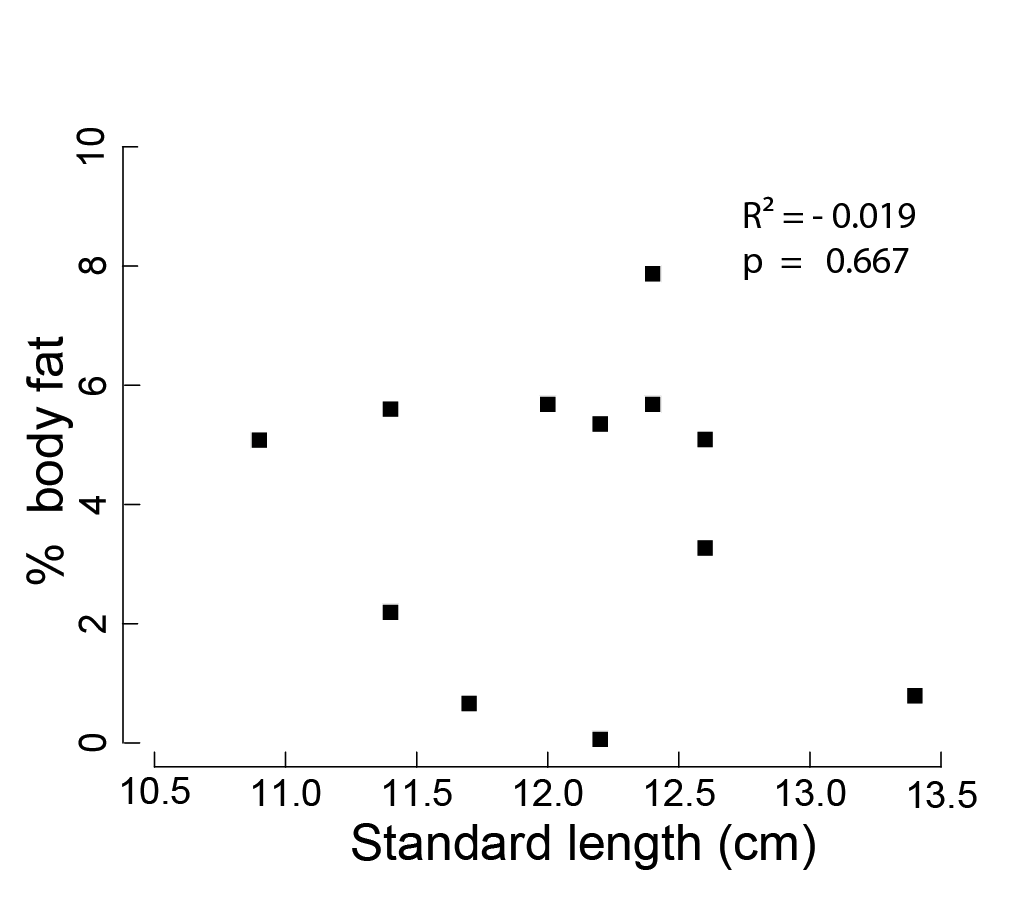
**

**Fig. S3:** Relationship of total fat stores (including evisceral and visceral fat) of nest males (N=12), measured in percent of body mass with body size (standard length; cm)
